# Real-world description of patients with resected epidermal growth factor receptor mutation positive non-small cell lung carcinoma treated with adjuvant osimertinib in an early access program in Italy: the ELBA observational study

**DOI:** 10.3389/fonc.2026.1724019

**Published:** 2026-02-16

**Authors:** Giulia Pasello, Claudia Proto, Carlo Genova, Alberto Pavan, Elisa Roca, Gabriele Minuti, Carmine D’Aniello, Raffaele Giusti, Sabrina Mariotti, Francesco Jacopo Romano, Valentina Polo, Lorenzo Belluomini, Editta Baldini, Francesca Mazzoni, Anna Cecilia Bettini, Valerio Gristina, Alessandro Russo, Francesco Grossi, Francesca Rita Ogliari, Francesco Gelsomino, Rita Chiari, Emilio Bria, Iacopo Fioroni, Diego Cortinovis, Elio Gregory Pizzutilo, Anna Zanetti, Giuseppe Pasqualetti, Barbara Roncari, Lucia Simoni, Annamaria Catino, the ELBA Study Group, Stefano Zanini, Vanessa Ruzzarin, Mattia De Nuzzo, Elena Tosoni, Giorgio Madonia, Valentina Marino, Viola Cutifani, Sara Pilotto, Jessica Insolda, Marco Sposito, Ilaria Francesca Furfaro, Alessia Longo, Arianna Fabbri, Davide Mannini, Alice Lunghi, Lorenzo Antonuzzo, Francesca Zepponi, Enrico Caliman, Alice Bardocci, Lucia Bonomi, Salvatore Intagliata, Laura Ghilardi, Silvia Moroni, Monica Gesualdo, Vincenzo Gennusa, Veronica Franchina, Paola Muscolino, Federico Cappuzzo, Vittoria Iorio, Eva Mazzotti, Mirella Moro, Giusy Parisi, Cristiano Serci, Rosa Maria Di Mauro, Marta Brambilla, Alessandra Carbone, Ilaria Vallini, Nicola Fogale, Maria Grazia Viganò, Roberto Viganò, Alessandra Bulotta, Michele Ferrara, Sara Oresti, Giovanna Marrapese, Stefano Stabile, Francesca Martinelli, Francesca Sperandi, Andrea De Giglio, Claudia Fusco, Serena Cosentino, Giulia Marcantognini, Eleonora Valli, Mattia Banfi, Vincenzo Montesarchio, Silvia Bianco, Ivana Caprice, Alessandro Contaldi, Giuseppe Viscardi, Antonietta Guida, Rosaria Laudiero, Manuela Otero, Raffaella Stefania Siesto Andrea Planeta, Giulia Barletta, Linda Zinoli, Maria Caterina Russo, Alessandro Scala, Susanna Yedro, Roberto Ginesi, Teresa Ginesi

**Affiliations:** 1 Department of Surgery Oncology and Gastroenterology, University of Padova, Padua, Italy; 2 Oncology 2, Istituto Oncologico Veneto IRCCS, Padua, Italy; 3 Istituto Nazionale Tumori, Milan, Italy; 4 Ospedale Policlinico San Martino, Genoa, Italy; 5 Department of Medical Oncology, AULSS 3 Serenissima, Ospedale dell'Angelo, Venezia, Italy; 6 Thoracic Oncology - Lung Unit, Pederzoli Hospital, Peschiera del Garda, Verona, Italy; 7 Clinical Trials Unit - Phase 1 and Precision Medicine, IRCCS National Cancer Institute Regina Elena, Rome, Italy; 8 Unit of Oncology, A.O. dei Colli P.O. Monaldi, Naples, Italy; 9 A.O.U. Sant'Andrea, Rome, Italy; 10 Policlinico Tor Vergata, Rome, Italy; 11 A.O.R.N. Cardarelli, Naples, Italy; 12 Oncology Unit, AULSS 2 Marca Trevigiana, Ca’ Foncello Hospital, Treviso, Italy; 13 Section of Oncology, Department of Engineering for Innovation Medicine (DIMI), University of Verona School of Medicine and Verona University Hospital Trust, Verona, Italy; 14 Ospedale San Luca, Lucca, Italy; 15 Oncology Department, Careggi University Hospital, Florence, Italy; 16 ASST Papa Giovanni XXIII, Bergamo, Italy; 17 Department of Precision Medicine in Medical, Surgical and Critical Care, AOUP Paolo Giaccone University Hospital, Palermo, Italy; 18 A.O. Papardo, Messina, Italy; 19 Department of Medicine and Technological Innovation, University of Insubria, Varese, Italy; 20 Medical Oncology Division, ASST Sette Laghi, Varese, Italy; 21 Dept. Medical Oncology, IRCCS Ospedale San Raffaele, Milan, Italy; 22 Università Vita-Salute San Raffaele, Pesaro (PU), Milan, Italy; 23 Medical Oncology, IRCCS Azienda Ospedaliero-Universitaria di Bologna, Bologna, Italy; 24 Ospedale San Salvatore - Muraglia, Pesaro (PU), Italy; 25 Fondazione Policlinico Universitario A. Gemelli, Rome, Italy; 26 Policlinico Universitario Campus Bio-Medico, Rome, Italy; 27 SC Medical Oncology, Fondazione IRCCS San Gerardo dei Tintori, Monza, Italy; 28 Department of Medicine, University of Milano-Bicocca, Milan, Italy; 29 ASST GOM Niguarda, Milan, Italy; 30 AstraZeneca Italy, Milan, Italy; 31 IQVIA Solutions Italy SRL, Modena, Italy; 32 Thoracic Oncology Unit, IRCCS Istituto Tumori "Giovanni Paolo II", Bari, Italy

**Keywords:** adjuvant treatment, EAP, early-stage NSCLC, EGFR, osimertinib, real-world

## Abstract

**Background:**

Following the ADAURA study results, showing that adjuvant osimertinib was associated with significant improvement in disease-free survival among patients with stage IB-IIIA epidermal growth factor receptor mutation positive (EGFRm+) non-small cell lung carcinoma (NSCLC), an Early Access Program (EAP) was activated in Italy to provide preapproval access to osimertinib.

**Methods:**

The ELBA observational retrospective cohort study aims to describe the characteristics, diagnostic workup, mutation testing, and treatment patterns of the patients included in the ADAURA EAP. The retrospective observation period was from the day of the first procedure leading to the pathological diagnosis of NSCLC (index date) to osimertinib initiation and data were obtained from medical records or other original documents available at the sites.

**Results:**

Overall, 71 patients were evaluable, mainly females (73.2%), with mean (SD) age of 67.5 (8.7) years. Age-adjusted Charlson Comorbidity Index scored 2 or 3 for 74.7% of patients without considering lung cancer. Forty-six (66.7%) out of 69 evaluable patients with available data were discussed at the multi-disciplinary team meeting. The median (25^th^-75^th^ percentiles) time from the initial diagnostic suspicion to the index date was 52.0 (30.0-67.0) days and from index date to EGFR test prescription 20.0 (0.0-42.0) days. Among patients with available data (N = 69), the tests were mostly single-gene polymerase chain reaction mutation-specific test (63.8%) and next-generation sequencing (33.3%). Primary tumor surgery was mostly lobectomy (n/N=63/71, 88.7%). Pathological staging was IB for 21.1% of patients, II for 43.7% and III for 35.2%. Adjuvant chemotherapy prior to osimertinib was administered in 32.4% of patients. Osimertinib was started after a median (25^th^-75^th^ percentiles) time from tumor resection of 2.9 (2.1-4.9) months.

**Conclusions:**

The ELBA Study showed an evolving landscape in biomarker-driven and molecular targeted therapies in early-stage NSCLC management towards the integration of mutational testing into clinical practice, with a growing focus on an optimal definition of adjuvant treatment.

## 1 Introduction

Lung cancer is one of the most common malignancies and causes of cancer-related death worldwide (1, 2). Non‐small cell lung cancer (NSCLC) accounts for 80-85% of all newly diagnosed lung cancer cases (3). About 20% of NSCLC patients are diagnosed with stages I and II, and 30% with stage III. Stages I-III are considered early to locally advanced stages for which the mainstay treatment is surgery, possibly within a multidisciplinary strategy including neo/adjuvant systemic treatment to reduce the risk of post-operative recurrences (4, 5). The past decade has seen new advancements in the management of NSCLC with remarkable progress in screening, diagnosis and treatment (6–8), highlighting the importance of individualized approaches and changing the landscape of post-operative adjuvant treatments (9–11). A subset of NSCLC patients harbors genetic alterations identified as driver mutations, such as epidermal growth factor receptor (EGFR) mutations, observed in approximately 20% of patients with NSCLC (12). The most common mutations in patients with EGFR mutation-positive (EGFRm+) NSCLC are deletions in exon 19 (Ex19del) or exon 21 L858R point mutation (12). Among new treatment strategies for these patients, the third-generation oral EGFR-tyrosine kinase inhibitor (TKI) osimertinib has emerged as an effective adjuvant therapy after complete tumor resection (13–15). The ADAURA study (ClinicalTrials.gov identifier: NCT02511106) showed that adjuvant osimertinib was associated with significant improvement in disease-free survival and overall survival among patients with stage IB to IIIA (as classified according to American Joint Committee on Cancer [AJCC] Cancer Staging Manual 7^th^ edition) EGFRm+ NSCLC (13–16). Following approval by the Food and Drug Administration (FDA) and the European Medicines Agency (EMA) (17), this regimen became the standard of care as adjuvant therapy for patients with completely resected stage IB-IIIA EGFRm+ NSCLC, according to the ESMO (18) and the National Comprehensive Cancer Network (NCCN) Clinical Practice Guidelines in Oncology (4).

In Italy, following the ADAURA study, an Early Access Program (EAP) was activated in March 2021 to provide pre-approval access to adjuvant osimertinib for patients with NSCLC with common EGFR sensitizing mutations after complete tumor resection (D5161R00036 EAP, hereinafter “ADAURA EAP”), which included 182 subjects across 82 cancer centers. While randomized clinical trials have demonstrated significant efficacy and a favorable safety profile of osimertinib in early NSCLC, there is a paucity of Italian real-world data on the use of osimertinib in the clinical practice (19, 20). In particular, we were specifically interested in deeply analyzing the population included in the ADAURA EAP, a program which brought the ADAURA protocol into real-world oncology clinical practice, with the aim to provide valuable data from different patient groups not routinely included in clinical trials, especially in this setting characterized by a heterogeneous patient population and post-operative approaches based on pathological findings and initial tumor burden.

The ELBA observational study aimed to describe the characteristics, diagnostic workup, mutation testing, and treatment patterns of patients diagnosed with EGFRm+ NSCLC treated with adjuvant osimertinib in the real-world Italian practice within the ADAURA EAP.

## 2 Materials and methods

### 2.1 Study design and data collection

ELBA was an Italian, multicenter, observational retrospective cohort study. For each included patient, the observation period started from the day of the first procedure that led to the pathological diagnosis of NSCLC, defined as “index date”. The retrospective observation period was framed between the index date and the date of initiation of the osimertinib treatment in the context of the ADAURA EAP (**Supplementary Figure S1**).

The data relating to the observation period were secondary data obtained from medical records or other original documents available at the sites and were collected using an electronic Case Report Form (eCRF). Collected variables are listed in **Supplementary Table S1**.

### 2.2 Study objectives

The primary objective was to describe the demographic and clinical characteristics of patients diagnosed with EGFRm+ NSCLC eligible for adjuvant osimertinib in the ADAURA EAP in Italy.

Secondary objectives were to describe (i) the diagnostic work-up of EGFRm+ NSCLC patients according to the standard clinical practice in Italy; (ii) the mutation testing patterns among NSCLC patients; (iii) the treatment patterns of patients diagnosed with EGFRm+ NSCLC before osimertinib initiation in the EAP.

### 2.3 Patient population

Male or female patients aged ≥ 18 years at the time of inclusion, with histologically-confirmed diagnosis of EGFRm+ NSCLC classified as stage IB, II, or III following complete tumor resection, who had entered the ADAURA EAP and had received at least one osimertinib administration were included in the study, provided they (or their legally acceptable representatives) had signed the ELBA Study subject informed consent and privacy form. Patients had NSCLC with common sensitizing EGFR mutations (Ex19Del, L858R), either alone or in combination with other EGFR mutations including T790M. Deceased patients were not included, as were those with unavailable medical chart or clinical information regarding patients’ characteristics, diagnostic approaches and treatments received for NSCLC prior to the inclusion in the study.

### 2.4 Statistical methods

The sample size is defined according to feasibility considerations. Further information can be found in **Supplementary Table S2**. No formal hypotheses were pre-specified and statistical significance testing was not planned for this observational study with a descriptive aim.

Eligible subjects were defined as included subjects meeting all inclusion criteria and not meeting the exclusion criterion. Patients with missing data for one or more variables were not excluded from the study. Missing data was not replaced. Descriptive statistics were provided to report demographic and clinical characteristics of patients, including mean and standard deviation (SD), median and quartiles, absolute and relative frequencies, according to the type of considered variables.

All analyses were performed using SAS Enterprise Guide version 8.2 and SAS 9.4 (SAS Institute, Cary, NC, USA). Study design and conduct, data monitoring, eCRF setup, and statistical analyses were performed by IQVIA Solutions Italy SRL, on behalf of AstraZeneca.

### 2.5 Ethics

The ELBA Study was approved by the Ethics Committees of all participating institutions, and was conducted in accordance with the Helsinki Declaration of 1964, and its later amendments.

## 3 Results

### 3.1 Patients’ demographic and clinical characteristics

From May 30, 2023, to March 21, 2024, a total of 73 patients were enrolled in the study across 26 Italian oncology sites willing to participate, provided each had included at least 3 patients in the EAP. Of these, 71 (97.3%) fulfilled all inclusion/exclusion criteria and were considered eligible and evaluable for statistical analysis (**Supplementary Figure S2**). Median (25^th^-75^th^ percentiles) duration of observation was 3.4 (2.7-5.8) months. Eligible patients were mainly females (n=52 patients, 73.2%) and, regarding ethnic background, 70 (98.6%) patients were White and one patient was Asian. Their demographic and clinical characteristics are detailed in **Table 1**. Overall, tobacco users were 36 (50.7%), specifically they were all cigarette smokers. Considering environmental exposure, 32 (45.1%) patients lived in small cities, villages, or rural areas, 22 (31.0%) in medium-sized cities (<500,000 inhabitants), and 17 (23.9%) in large cities (500,000 inhabitants or more).

**Table 1:** Demographic and clinical characteristics of eligible patients (N = 71).

Baseline characteristics	n (%)
Sex assigned at birth, females	57 (73.2)
Mean (SD) age at the index date (years)	67.5 (8.7)
Tobacco users	36 (50.7)
Previous users	25 (35.2)
Current users	11 (15.5)
Patients with family history of lung cancer in any first-degree relative (parent, sibling, or child)^a^	6 (15.0)
Patients with ≥1 sign or symptom at the index date^b^	17 (23.9)
Loss of weight	6 (8.5)
Dyspnea	5 (7.0)
Chest pain	4 (5.6)
Dysphonia	3 (4.2)
Cough	2 (2.8)
Dizziness	2 (2.8)
Hemoptoe, altered kinesthesia, pneumonia, right shoulder pain, each	1 (1.4)
WHO-PS	
0	52 (73.2)
1	19 (26.8)
Age-adjusted CCI score at the index date (patients per score)^c^	
0	3 (4.2)
1	7 (9.9)
2	23 (32.4)
3	30 (42.3)
4	6 (8.5)
5	2 (2.8)

CCI, Charlson Comorbidity Index; Index date, day of the first procedure that led to the pathological diagnosis of NSCLC; NSCLC, Non-Small Cell Lung Cancer; SD, Standard Deviation; WHO-PS, World Health Organization – Performance Status.

a The percentage was computed on the N = 40 patients with available data. Five patients had one first-degree family member affected by lung cancer, and one patient had three.

b More than one option could have been recorded per patient.

c The age-adjusted CCI has been calculated excluding active localized NSCLC from the scoring.

Only 17 (23.9%) eligible patients had at least one sign detected at physical examination or one symptom reported at the index date (details in **Table 1**). At the index date, the median (25^th^-75^th^ percentiles) age-adjusted Charlson Comorbidity Index (CCI) score without considering current lung cancer was 3.0 (2.0-3.0), with 53 patients (74.6%) scoring 2 or 3 (details in **Table 1**). Eligible patients with medical-surgical history other than NSCLC were 59 (83.1%), in 49 of which (69.0%) the condition was ongoing at the index date (details in **Supplementary Table S3**). Also at the index date, 35 (49.3%) patients were being treated with at least one drug for other non-NSCLC related conditions (details of medications in **Supplementary Table S3**).

The most common location of the primary tumor was peripheral (n=44 patients, 62.9%), while 26 (37.1%) patients had central NSCLC (one patient with missing data). **Table 2** summarizes the overall clinical cancer stage at the index date according to the AJCC Cancer Staging Manual 8^th^ edition. At postoperative histological examination, all NSCLC but one (n=70, 98.6%) were adenocarcinomas. Patients with no regional lymph nodes involvement at postoperative assessment were 32 (45.1%). **Table 2** also shows the postoperative pathological cancer staging and the node classification among patients with stages II and III (AJCC Cancer Staging Manual 8^th^ edition).

**Table 2:** Clinical and pathological cancer staging (AJCC Cancer Staging Manual 8th edition).

NSCLC cancer staging		n (%)
Overall clinical cancer staging at the index date^a^		
	I	29 (41.4)
	II	27 (38.6)
	III	14 (20.0)
	Missing	1
Detailed clinical cancer staging at the index date^a^		
	IA1	1 (1.4)
	IA2	2 (2.9)
	IA3	7 (10.0)
	IB	19 (27.1)
	IIA	8 (11.4)
	IIB	19 (27.1)
	IIIA	13 (18.6)
	IIIB	1 (1.4)
	Missing	1
Detailed pathological cancer staging (postoperative)^a^		
	IB	15 (21.1)
	IIA	4 (5.6)
	IIB	27 (38.0)
	IIIA	24 (33.8)
	IIIB	1 (1.4)
N classification among patients with pathological cancer stage II^b^		
	N0	16 (51.6)
	N1	15 (48.4)
N classification among patients with pathological cancer stage III^c^		
	N0	1 (4.0)
	N1	3 (12.0)
	N2	21 (84.0)

AJCC, American Joint Committee on Cancer; Index date: day of the first procedure that led to the pathological diagnosis of NSCLC; NSCLC, Non-Small Cell Lung Cancer.

a Percentages were computed excluding patients with missing values from the total.

b Percentages were computed over the total number of patients with pathological cancer stage II (N = 31).

c Percentages were computed over the total number of patients with pathological cancer stage III (N = 25).

### 3.2 Diagnostic work-up

The diagnosis of NSCLC was made in 2021 in 65 (91.5%) patients, in 2020 in 5 (7.0%), and in 2022 in one patient. The initial suspicion of lung cancer came from the incidental detection of a nodule in 42 (59.2%) patients, from symptomatic disease at presentation in 20 (28.2%), and for other reasons in 6 (8.5%) cases (3 patients with missing data). The median (25^th^-75^th^ percentiles) time interval from the initial suspicion of lung cancer to the index date was 52.0 (30.0-67.0) days. Most patients (n=48, 67.6%) underwent the diagnostic process entirely in the clinical site where the patient was included in the ELBA Study, while 23 (32.4%) did not. All patients underwent at least one imaging evaluation performed during the whole NSCLC diagnostic process, as detailed in **Table 3**. Outside of surgical resection, another 43 (60.6%) eligible patients had at least one cyto-histopathologic examination during the NSCLC diagnostic work-up, as shown in **Table 3**. Lymph node preoperative sampling was performed during the diagnostic process in 18 (25.4%) patients, while bronchoscopy was performed in 22 (31.0%) patients.

**Table 3:** Type of imaging and cyto-histopathologic examinations during the NSCLC diagnostic process.

NSCLC diagnostic process		n (%)
Type of imaging examination^a^		
	FDG-PET scan	59 (83.1)
	CT scan, chest	53 (74.6)
	CT scan, brain	34 (47.9)
	Bronchoscopy	22 (31.0)
	X-ray, chest	18 (25.4)
	EBUS	9 (12.7)
	CT scan, total body	7 (9.9)
	MRI, brain	4 (5.6)
	MRI, abdomen	1 (1.4)
	Bone scan	1 (1.4)
Type of pathological examination^a^		
	Transbronchial needle aspiration	13 (18.3)
	Core-needle biopsy	9 (12.7)
	Fine-needle aspiration	7 (9.9)
	Percutaneous needle biopsy	7 (9.9)
	Transbronchial lung biopsy	5 (7.0)
	Thoracotomy biopsy	5 (7.0)
	Bronchial alveolar lavage	3 (4.2)
	Thoracentesis	1 (1.4)
	Thoracoscopy, diagnostic	1 (1.4)
	Mediastinoscopy biopsy	1 (1.4)
	Other types of evaluation	6 (8.5)

CT, Computed Tomography; EBUS, Endobronchial Ultrasound; FDG, Fluorodeoxyglucose; MRI, Magnetic Resonance Imaging; NSCLC, Non-Small Cell Lung Cancer; PET, Positron Emission Tomography.

a Patients may have undergone more than one examination.

In the diagnostic process, the multi-disciplinary team (MDT) was involved in 46 (66.7%) cases with available data while it was not involved in 23 (33.3%) cases (2 patients with missing data). Specialists consistently included in the MDT were medical oncologists (100% of cases), thoracic surgeons and pathologists (97.8%), followed by radiation oncologists (76.1%), radiologists (65.2%), pulmonologists/bronchoscopists (54.3%), molecular biologists (43.5%), and nuclear medicine physicians (34.8%), while in 2 cases an anesthetist was also involved.

Stratifying the MDT involvement in the diagnostic process by NSCLC clinical staging at the index date (AJCC Cancer Staging Manual 8^th^ edition), such involvement was most frequent in stages III (n/N=11/14; 78.6%), and II (n/N=19/25; 76.0%). The MDT was involved in the indication to tumor resection in 41 (58.6%) cases. In these 41 patients, the MDT participated in the assessment of all anatomical, biological, and functional operability criteria in accordance with the AIOM Guidelines 2020 on lung cancer (21). The most involved specialists were medical oncologists (n=41; 100%), thoracic surgeons and pathologists (n=40; 97.6%), followed by radiation oncologists (n=30; 73.2%) and radiologists (n=28; 68.3%).

### 3.3 Mutation tests

The EGFR mutation testing was most frequently prescribed by the medical oncologist (43 of 46 patients managed by the MDT in the diagnostic process, 93.5%) or performed by the pathologist as reflex test (34 patients, 73.9%).

The median (25^th^-75^th^ percentiles) time interval from the index date to the date of initial EGFR test prescription (regardless of pre-operative or surgical sample) was 20.0 (0.0-42.0) days, and from initial prescription to the date of the report of the test result was 16.0 (8.0-27.0) days. The type of EGFR test was mostly a single-gene polymerase chain reaction (PCR) mutation-specific test (n=44; 63.8%), followed by next-generation sequencing (NGS) on NGS-based platform (n=23; 33.3%). An automated PCR-based platform was used only in 2 (2.9%) cases with available data (2 patients with data missing). The biologic samples tested were surgical samples from tumor resection in 58 (81.7%) cases and preoperative biopsy samples in 9 (12.7%) cases. Preoperative and surgical samples were both analyzed in 2 (2.8%) cases, as well as preoperative cytological samples. The type of EGFR mutation detected before osimertinib treatment was Ex19Del in 44 (62.0%) patients, L858R in 28 (39.4%) patients, and T790M in one patient, with more than one mutation recorded per patient. Other mutation tests other than EGFR were performed before osimertinib treatment in 47 (66.2%) patients, as specified in **Table 4**. Programmed death-ligand 1 (PD-L1) level was tested before osimertinib treatment in 41 (57.7%) of 71 eligible patients. The Tumor Proportion Score (TPS) value was <1% in 16 (39.0%) patients, between 1% and 49% in 24 (58.5%) patients, and ≥50% in one patient with PD-L1 level tested.

**Table 4:** Mutational tests (other than EGFR) performed in the NSCLC diagnostic process.

Other mutational tests		n (%)
Patients with mutational tests (other than EGFR) performed before osimertinib treatment initiation		47 (66.2)
Genes tested^a^		
	ALK	41 (87.2)
	ROS1	37 (78.7)
	BRAF	33 (70.2)
	KRAS	31 (66.0)
	MET	21 (44.7)
	RET	21 (44.7)
	HER2	9 (19.1)
	NTRK (1,2,3)	4 (8.5)
	Other genes tested*, n*	5 (10.6)
	*NRAS*	4
	*PIK3CA*	3
	*FGFR3*	1
	*IDH1*	1
	*IDH2*	1
	*KIT*	1
	*PDGFRA*	1

EGFR, epidermal growth factor receptor; NSCLC, Non-Small Cell Lung Cancer.

a Percentages were computed on eligible patients undergone mutation testing procedures (other than EGFR mutational tests) before osimertinib treatment initiation in the context of the ADAURA EAP (N = 47).

### 3.4 Treatment patterns

The mean (SD) time from index date to tumor resection was 0.8 (1.3) months. Primary tumor surgery was mostly lobectomy (n/N=63/71; 88.7%). Bilobectomy was performed in additional 5 (7.0%) cases and total pneumonectomy, wedge resection, and parietal pleurectomy in 1 case each. On lymph nodes, systematic sampling was performed in 16 (22.5%) patients, and random sampling in 4 (5.6%). Mediastinal lymph node dissection was performed in 27 (38.0%) patients, lobe-specific systematic node dissection in 8 (11.3%). The remaining patients underwent other types of surgical interventions, such as extended lymph node dissection. The most common type of surgical approach was video-assisted thoracic surgery (VATS) (n/N=42/71; 59.2%), followed by open surgery (n=23; 32.4%), and robotically-assisted thoracic surgery (RATS) (n=6; 8.5%). Adjuvant chemotherapy prior to osimertinib was performed in 23 of the 71 (32.4%) eligible patients (as detailed in **Table 5**). Thirteen of these patients had pathological stage III, and 10 had stage II (9 of whom were IIB). Overall, osimertinib was started after a median (25^th^-75^th^ percentiles) time from tumor resection of 2.9 (2.1-4.9) months.

**Table 5:** Adjuvant chemotherapy regimens administered before osimertinib.

Adjuvant chemotherapy		n (%)
Patients treated with adjuvant chemotherapy following surgery		23 (32.4)
Types of chemotherapy administered in the adjuvant setting^a,b^		
	Cisplatin	19 (82.6)
	Vinorelbine	15 (65.2)
	Gemcitabine	8 (34.8)
	Carboplatin	5 (21.7)
	Pemetrexed	1 (4.3)
	*Chemotherapy combinations, n*	
	*Cisplatin, Vinorelbine*	13
	*Cisplatin, Gemcitabine*	6
	*Carboplatin, Gemcitabine*	2
	*Carboplatin, Vinorelbine*	2
	*Carboplatin, Pemetrexed*	1
Reasons for adjuvant chemotherapy permanent discontinuation^a,b^		
	Treatment regimen completed as per planning	18 (78.3)
	Unacceptable toxicity	5 (21.7)
	No overall value of continued treatment regimen according to medical judgement	1 (4.3)

a Percentages were computed on eligible patients treated with adjuvant chemotherapy following surgery (N = 23).

b More than one option could have been recorded per patient.

## 4 Discussion

Description of patients’ demographic and clinical characteristics was the primary study objective. The ELBA patients (N = 71) had a mean age of 68 years and, as shown in the ADAURA trial (13), were mainly females, which is consistent with the higher reported prevalence of EGFR mutations in women (22, 23). WHO Performance Status (PS) was good – 0 for 73% of patients and 1 in the remaining 27% – consistently with the fact that patients were all fit for surgery.

A high percentage of patients (83%) had at least one other relevant medical condition occurred in the past and/or ongoing at NSCLC diagnosis, including a high percentage of other solid organ malignancies (27% of the overall participants), higher than that reported in a large patient population with advanced metastatic NSCLC (24), where about 80% of patients had no other tumors. Consistently, the age-adjusted CCI score – calculated at the index date – was quite high, with 42% of patients having a CCI score of 3 and 32% having a CCI score of 2. The CCI has been reported as a better prognostic factor than individual comorbid conditions in NSCLC (25, 26) and these high scores, in addition to the high mean age, describe an overall clinically complex population.

NSCLC histotype was adenocarcinoma in all patients but one, in line with the higher prevalence of EGFR mutations in lung adenocarcinoma than in non-adenocarcinomas (22). Most patients were in pathological stage IIB and IIIA (38% and 34%, respectively), but about 21% of patients were in stage IB for which adjuvant chemotherapy is not recommended – except in selected cases (27, 28) – and targeted therapy represents a major treatment opportunity. It is worth noting that 10 (14.3%) patients with available data (N = 70) were classified as stage IA (AJCC Cancer Staging Manual 8^th^ edition) at clinical evaluation while at pathological evaluation on surgical sample they were evaluated as stages IB-IIIA. This is in line with a previous report that showed a possible clinical stage underestimation, especially in very early stages of NSCLC (29).

Overall, an MDT was involved in only two thirds of the patients, which is lower than currently expected, also considering that recent data confirmed that MDT-based NSCLC management is associated with better quality of care and even longer overall survival (30). However, it is interesting to look at MDT involvement stratified by the different stages of the disease: most of the cases not managed by an MDT (57%) were in the earliest clinical stages of the diseases, IA and IB. It can be hypothesized that patients at the lowest stages were referred simply and directly to surgery, without convening the MDT. Interestingly, the radiation oncologist was frequently involved in the MDT, which is consistent with the continuous improvements in radiotherapy and recent developments in the field of individualized radiotherapy also in early-stage NSCLC (31).

The diagnostic work-up was carried out for most patients in the same year (2021) and in the same clinical site, ensuring a good uniformity of approaches. It is worth noting that the percentage of brain CT as staging procedure was unexpectedly low, considering that the incidence of brain metastases in patients with EGFR-mutant NSCLC has been estimated to be up to 70% (32–35). A possible explanation could be that during the pre-surgery diagnostic process the results of the EGFR tests were not yet available, with 41.4% of patients with available data (N = 70) classified as having clinical staging I at the index date. Also noteworthy is the low percentage of patients who underwent pre-operative lymph node sampling, a procedure that should normally be performed in patients with positive lymph nodes (N1 and N2) on clinical-radiological assessment (18). The limited median interval – of only 20 days – between the index date and the prescription of the EGFR test is a positive note, which confirms the growing practice of carrying out mutational tests even in the early stages of NSCLC. The recent CancerLinQ Discovery database retrospective analysis reported that the frequency of EGFR testing tended to increase with increasing severity of NSCLC disease stage, despite – as the authors underline – biomarker testing is important to ensure appropriate therapies and optimal outcomes for all patients with stage I–III NSCLC (36). On the other hand, recent data from a real-world, unselected cohort of lung adenocarcinoma have shown that the percentage of patients harboring EGFR mutations is relatively high also in the lower disease stages, providing a strong rationale for routine testing of early stage lung cancers for EGFR mutations in the West-European population (37). The Ex19del and L858R mutations represent approximately 85%-90% of EGFR mutations in NSCLC (38) and were consistently present in almost all ELBA patients. The most used method for EGFR testing was the specific single-gene PCR test (64% of cases), followed by the NGS-based platform technique, both recognized as acceptable for detecting EGFR mutations, although NGS is believed to achieve higher sensitivity values than PCR-based methods (39). In our population, the test was performed on the surgical specimen in the majority of patients (85% overall), while currently preoperative biopsies are usually performed. Interestingly, two thirds of our patients underwent other mutation tests, which we consider encouraging especially referred to 2021. The tested genes – ALK, ROS1, BRAF, KRAS, MET, RET – are in line with recent literature (10). On the other hand, the percentage of patients tested for PD-L1 was unexpectedly low, considering that NSCLCs are usually subjected to the analysis of PD-L1 protein expression to guide the use of immune checkpoint inhibitors (40). Even more surprising were the reported percentages of tumor cells expressing PD-L1, with only one patient expressing PD-L1 in ≥50% of cells, in contrast with previously published data reporting PD-L1 as overexpressed in NSCLC (41–43).

The most common type of surgical approach was VATS lobectomy, which is indeed believed to be becoming the standard of care for early-stage NSCLC (1). Italy is recognized as having always been at the forefront of minimally invasive surgery, particularly in the field of thoracic surgery, with more than 95% of Italian thoracic surgery units participating in the VATS Group registry, in which more than 12000 cases are registered (44). Following surgery and before receiving osimertinib within the ADAURA EAP, less than half (41%) of patients with pathological stage II-III received adjuvant chemotherapy after a median time of about 2 months, which consisted predominantly of four cycles of cis/carboplatin-based regimens in line with existing literature (15). Alongside the pathological stage, the presence of medical conditions other than NSCLC in a substantial proportion of patients might have contributed to influencing the choice of adjuvant treatment approach (45), although the study protocol did not foresee collecting the clinical reasons for administering or withholding adjuvant chemotherapy.

The ELBA Study has some other limitations. First, the retrospective data of patients enrolled in the EAP cannot fully represent the current Italian standard of diagnosis and care also considering that the participating study sites were not randomly sampled but chosen according to their participation in the ADAURA EAP. Although not all clinical institutions activated in the EAP were involved in the ELBA Study, the relatively high number of participating sites distributed throughout Italy can be considered a mitigating factor for this limitation. Furthermore, as in all retrospective studies, there was the risk of having a proportion of missing data. However, since the availability of medical charts or clinical information on treatments received for NSCLC was an eligibility criterion, the risk of missing data for relevant clinical information was minimized. In addition, as a mitigation preventive action, a feasibility study assessment allowed us to evaluate if specific variables were systematically missing or not available in standard clinical practice, which permitted to define the expectation of key variables availability. Another limitation is the exclusion from the study of dead or untraceable subjects. However, since the study included subjects with early-stage disease who underwent radical surgery, we believe that the exclusion of those who died prematurely may have had a limited impact both in terms of selection bias and on the achievement of the study objectives. Based on the literature, it was expected that over 95% of target subjects potentially involved in the EAP would have been alive during the enrollment period of the ELBA Study, as specified in the study protocol, and the updated results of the ADAURA study confirm this assumption (15). Regarding traceability, since all patients were included in the EAP activated in specific clinical centers, the percentage of untraceable subjects is likely negligible, also thanks to the short enrollment period. Lastly, the study lacks sex-stratified analyses, because the limited number of subjects, especially males, discouraged us from performing a subgroup analyses. Nevertheless, we recognize this may represent a limitation to our results’ generalizability.

## 5 Conclusions

Despite the above-listed limitations, the ELBA Study provided an insight into the characteristics, the diagnostic work-up, the mutation testing, and treatment patterns of patients diagnosed with EGFRm+ NSCLC and treated with adjuvant osimertinib following complete tumor resection in a real-world setting in Italy. The results show a rapidly evolving landscape in biomarker-driven and molecular targeted therapies in NSCLC management towards the integration of biomarkers and mutational testing into clinical practice. Moreover, there seems to be a growing focus on an optimal definition of adjuvant treatment, based on a personalized treatment paradigm to reduce recurrence risk. Finally, the importance of the involvement of MDT in the early diagnosis and treatment of NSCLC seems to be recognized, although there is room for further improvement.

## Data Availability

The datasets generated during and/or analyzed during the current study are not publicly available due to the sensitive nature of the data collected in this study. Requests to access the datasets should be directed to serena.losi@astrazeneca.com.

## Group members of the ELBA Study Group

Stefano Zanini, Vanessa Ruzzarin, Mattia De Nuzzo (Istituto Oncologico Veneto IRCCS, Padua, Italy); Elena Tosoni, Giorgio Madonia (Pederzoli Hospital, Peschiera del Garda - Verona, Italy); Valentina Marino, Viola Cutifani (Ca' Foncello Hospital, Treviso, Italy); Sara Pilotto, Jessica Insolda, Marco Sposito (Verona University Hospital Trust, Verona, Italy); Ilaria Francesca Furfaro, Alessia Longo, Arianna Fabbri, Davide Mannini, Alice Lunghi (Ospedale San Luca, Lucca, Italy); Lorenzo Antonuzzo, Francesca Zepponi, Enrico Caliman, Alice Bardocci (Careggi University Hospital, Florence, Italy); Lucia Bonomi, Salvatore Intagliata, Laura Ghilardi, Silvia Moroni (ASST Papa Giovanni XXIII, Bergamo, Italy); Monica Gesualdo (IRCCS Istituto Tumori "Giovanni Paolo II", Bari, Italy); Vincenzo Gennusa (AOUP Paolo Giaccone University Hospital, Palermo, Italy); Veronica Franchina, Paola Muscolino (A.O. Papardo, Messina, Italy); Federico Cappuzzo, Vittoria Iorio, (IRCCS National Cancer Institute Regina Elena, Rome, Italy); Eva Mazzotti, Mirella Moro (A.O.U. Sant'Andrea, Rome, Italy); Giusy Parisi, Cristiano Serci (Policlinico Tor Vergata, Rome, Italy); Rosa Maria Di Mauro, Marta Brambilla (Istituto Nazionale Tumori, Milan, Italy); Alessandra Carbone (Fondazione IRCCS San Gerardo dei Tintori, Monza, Italy); Ilaria Vallini (ASST Sette Laghi, Varese, Italy); Nicola Fogale, Maria Grazia Viganò, Roberto Ferrara, Alessandra Bulotta, Michele Ferrara, Sara Oresti (IRCCS Ospedale San Raffaele, Milan, Italy); Giovanna Marrapese, Stefano Stabile, Francesca Martinelli (ASST GOM Niguarda, Milan, Italy); Francesca Sperandi, Andrea De Giglio, Claudia Fusco, Serena Cosentino (IRCCS Azienda Ospedaliero-Universitaria di Bologna, Bologna, Italy); Giulia Marcantognini, Eleonora Valli, Mattia Banfi (Ospedale San Salvatore – Muraglia (PU), Italy); Vincenzo Montesarchio, Silvia Bianco, Ivana Caprice, Alessandro Contaldi, Giuseppe Viscardi, Antonietta Guida, Rosaria Laudiero (A.O. dei Colli P.O. Monaldi, Naples, Italy); Manuela Otero, Raffaella Stefania Siesto Andrea Planeta (A.O.R.N. Cardarelli, Naples, Italy); Giulia Barletta, Linda Zinoli (Ospedale Policlinico San Martino, Genoa, Italy); Maria Caterina Russo, Alessandro Scala, Susanna Yedro (Fondazione Policlinico Universitario A. Gemelli, Rome, Italy); Roberto Ginesi, Teresa Grassani (Policlinico Universitario Campus Bio-Medico, Rome, Italy).
