# Supplementary figures and images for: Real-world description of patients with resected epidermal growth factor receptor mutation positive non-small cell lung carcinoma treated with adjuvant osimertinib in an early access program in Italy: the ELBA observational study
Source: Front Oncol. 2026 Feb 16;16:1724019. doi: 10.3389/fonc.2026.1724019 (PMC12951046; doi:10.3389/fonc.2026.1724019)

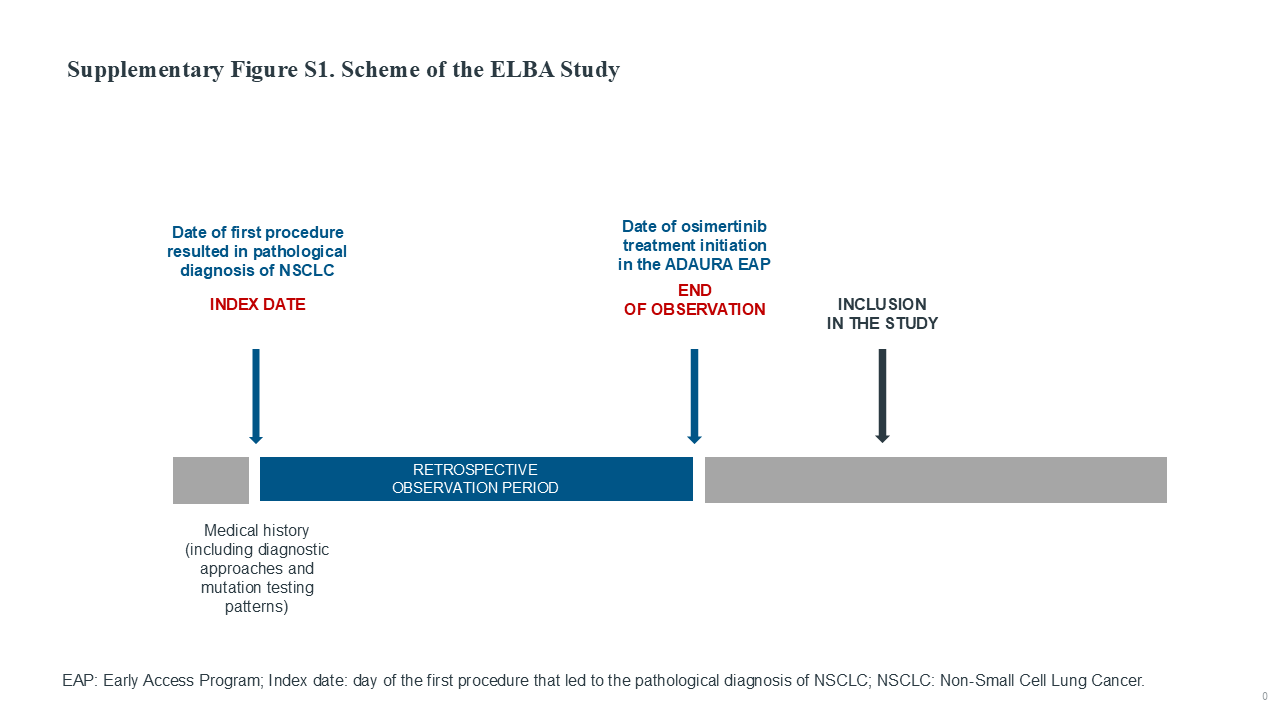

Supplement: Supplementary file 1 [file Image1.tif]

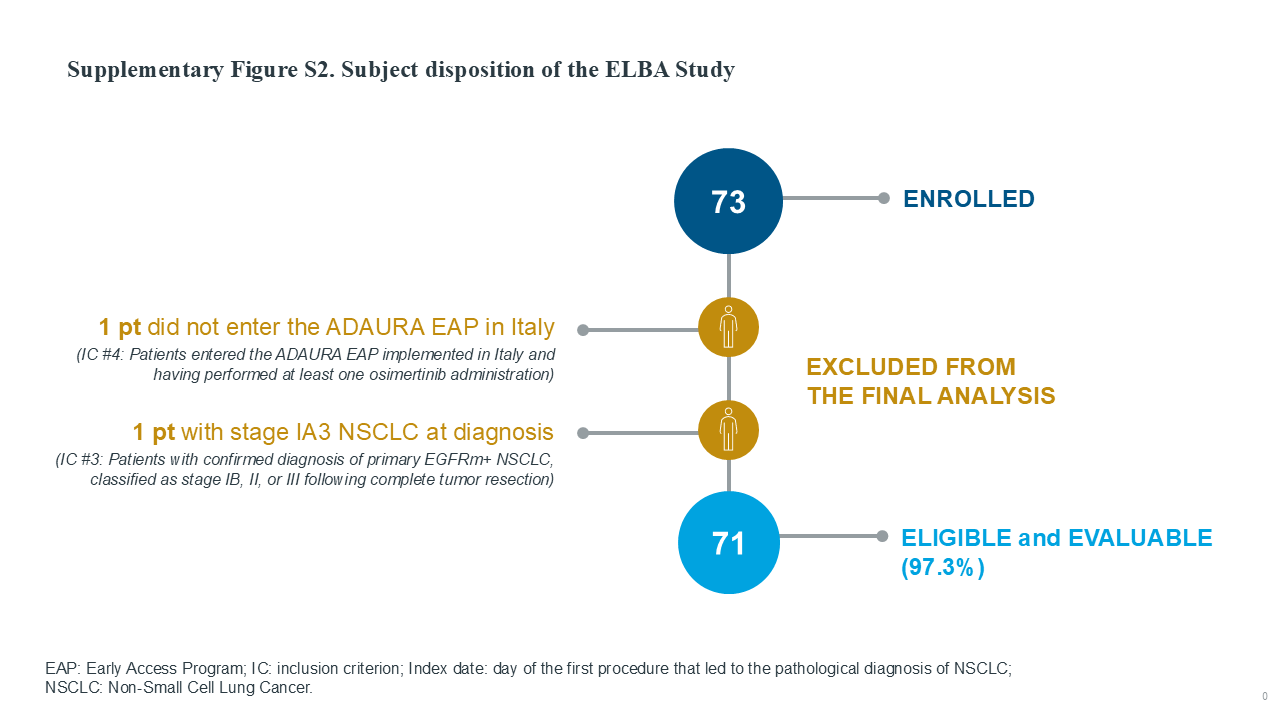

Supplement: Supplementary file 2 [file Image2.tif]
