# Supplementary material for: Real-world description of patients with resected epidermal growth factor receptor mutation positive non-small cell lung carcinoma treated with adjuvant osimertinib in an early access program in Italy: the ELBA observational study
Source: Front Oncol. 2026 Feb 16;16:1724019. doi: 10.3389/fonc.2026.1724019 (PMC12951046; doi:10.3389/fonc.2026.1724019)
Supplement: Supplementary file 3 [file Table1.docx]

Supplementary Material

Real-world description of patients with resected epidermal growth factor receptor mutation positive non-small cell lung carcinoma treated with adjuvant osimertinib in an early access program in Italy: the ELBA observational study

**Supplementary Table S1 – Variables collected in the ELBA Study**

|  | **TIME-POINTS** | |
| --- | --- | --- |
| **VARIABLES** | **Index date** | **Study inclusion** |
| **Inclusion procedures**  Date of study inclusion; inclusion/exclusion criteria; ELBA Study informed consent and privacy consent |  | X |
| **Demographics**  Age; gender; race/ethnicity | X |  |
| **Physical examination**  Weight, height; clinical signs and symptoms; WHO-PS | X |  |
| **Comorbidities and relevant medical-surgical history**  Type of relevant medical-surgical condition; medications ongoing at index date | X |  |
| **Risk factors**  Smoking habits, including exposure to environmental tobacco smoke; exposure to other chemical substances; main living place; family history of lung cancer | X |  |
| **Diagnostic process**  Initial diagnostic suspicion; types of evaluations during the diagnostic process (e.g., radiological/imaging examination, cyto-histopathologic examination); time interval from the initial diagnostic suspicion to the index date; MDT involvement in the diagnostic process, including MDT specialties | X |  |
| **Mutation testing and biomarkers**  Testing procedures’ details; type of driver gene mutation tested; type of biomarker tested | X | |
| **NSCLC characteristics**  Index date; primary tumor location; clinical cancer staging at index date, and pathologic (post-operative) TNM classification and cancer staging; regional lymph nodes involvement; histologic type | X |  |
| **Anticancer treatments for NSCLC**   - Tumor resection, including lymph node sampling or dissection: date of tumor resection; type of surgical procedure; outcome of resection and date of procedure. - Other (non-surgical) treatments: treatment setting; treatment type and regimen; drug name; regimen start and end date; reason for regimen discontinuation; date of osimertinib treatment initiation. | X | |

Index date: day of the first procedure that led to the pathological diagnosis of NSCLC; MDT: Multi-Disciplinary Team; NSCLC: Non-Small Cell Lung Cancer; TNM: Tumor Node, and Metastases; WHO-PS: World Health Organization - Performance Status.
