# Supplementary material for: Real-world description of patients with resected epidermal growth factor receptor mutation positive non-small cell lung carcinoma treated with adjuvant osimertinib in an early access program in Italy: the ELBA observational study
Source: Front Oncol. 2026 Feb 16;16:1724019. doi: 10.3389/fonc.2026.1724019 (PMC12951046; doi:10.3389/fonc.2026.1724019)
Supplement: Supplementary file 4 [file Table2.docx]

Supplementary Material

Real-world description of patients with resected epidermal growth factor receptor mutation positive non-small cell lung carcinoma treated with adjuvant osimertinib in an early access program in Italy: the ELBA observational study

**Sample size justification**

The sample size was defined according to feasibility considerations with respect to both the enrollment period duration and the number of participating sites. In addition, we assumed that up to 10% of the enrolled patients could not be evaluable for the primary analysis (e.g., due to failure in meeting the eligibility criteria or missing/non-available data). Therefore, considering a target sample size of 80, we expected 72 patients to be evaluable for the primary analysis.

Starting from the ADAURA study results (osimertinib arm)^1^ we calculated the 95% confidence interval (CI) estimations for the expected proportions of different patient characteristics of interest (Supplementary Table S2) for a sample size of 72, under the assumption that the CI is based on the normal approximation.^2^

**Supplementary Table S2 – 95% confidence interval (CI) estimations according to different patient characteristics.**

| **Evaluation** | **Expected proportion** | **95% CI limits** |
| --- | --- | --- |
| Sex assigned at birth = female | 68% | 57.2%-78.8% |
| Smoking history = yes | 32% | 21.2%-42.8% |
| WHO-PS = 0 | 64% | 52.9%-75.1% |
| Regional lymph nodes involvement = N0 | 41% | 29.6%-52.4% |

CI: Confidence Interval; WHO-PS: World Health Organization – Performance Status

# References

1. Wu YL, Tsuboi M, He J, John T, Grohe C, Majem M, et al; ADAURA Investigators. Osimertinib in Resected EGFR-Mutated Non-Small-Cell Lung Cancer. N Engl J Med. 2020;383(18):1711-23. https://doi:10.1056/NEJMoa2027071.

2. Dixon WJ, Massey Jr FJ. Introduction to Statistical Analysis. 4th Edition, New York, NY: McGraw-Hill; 1983.
