# Supplementary material for: Real-world description of patients with resected epidermal growth factor receptor mutation positive non-small cell lung carcinoma treated with adjuvant osimertinib in an early access program in Italy: the ELBA observational study
Source: Front Oncol. 2026 Feb 16;16:1724019. doi: 10.3389/fonc.2026.1724019 (PMC12951046; doi:10.3389/fonc.2026.1724019)
Supplement: Supplementary file 5 [file Table3.docx]

Supplementary Material

Real-world description of patients with resected epidermal growth factor receptor mutation positive non-small cell lung carcinoma treated with adjuvant osimertinib in an early access program in Italy: the ELBA observational study

**Supplementary Table S3 – Medical-surgical history other than NSCLC and concomitant medications (N=71 eligible patients)**

|  | | **n (%)** |
| --- | --- | --- |
| **Patients with at least one medical condition other than NSCLC occurred in the past and/or ongoing at the index date** |  | 59 (83.1) |
| **Medical conditions**^a,b^ |  |  |
|  | Hypertension | 32 (45.1) |
|  | Solid organ malignancy (other than NSCLC) | 19 (26.8) |
|  | Diabetes mellitus | 8 (11.3) |
|  | Dyslipidemia | 7 (9.9) |
|  | Hypercholesterolemia | 7 (9.9) |
|  | Osteoporosis | 7 (9.9) |
|  | Hypothyroidism | 5 (7.0) |
|  | Benign prostatic hyperplasia | 5 (7.0) |
|  | Chronic gastritis | 4 (5.6) |
|  | Depression | 4 (5.6) |
|  | COPD | 3 (4.2) |
|  | Other unspecified respiratory disease | 3 (4.2) |
|  |  |  |
| **Primary body site(s) affected by other solid organ malignancy^a,c^** |  |  |
|  | Breast | 5 (26.3) |
|  | Skin | 3 (15.8) |
|  | Uterus | 3 (15.8) |
|  | Colon-rectum | 2 (10.5) |
|  | Thyroid | 2 (10.5) |
|  | Bladder | 1 (5.3) |
|  | Kidney/Ureter | 1 (5.3) |
|  | Esophagus | 1 (5.3) |
|  | Thymus | 1 (5.3) |
|  | Lip | 1 (5.3) |
|  | Parotid | 1 (5.3) |
|  |  |  |
| **Solid organ malignancy (other than NSCLC) extension^c^** |  |  |
|  | Localized | 18 (94.7) |
|  | Metastatic | 1 (5.3) |
|  |  |  |
| **Patients treated with at least one medication (other than NSCLC treatment) ongoing at the index date** |  | 35 (49.3) |
|  |  |  |
| **Concomitant medications by drug class^a^** | Antihypertensives | 21 (29.6) |
|  | Proton pump inhibitors | 9 (12.7) |
|  | Statins | 9 (12.7) |
|  | Antithrombotic drugs | 8 (11.3) |
|  | Diabetes medications | 6 (8.5) |
|  | Hormone replacement therapies | 5 (7.0) |
|  | Antidepressants | 3 (4.2) |
|  | Analgesics | 2 (2.8) |
|  | Antiarrhythmics | 2 (2.8) |
|  | Anti-infective therapies | 1 (1.4) |
|  | Cancer therapies (for malignancies other than NSCLC) | 1 (1.4) |
|  | Diuretics | 1 (1.4) |
|  | NSAIDs | 1 (1.4) |
|  | Other drug class | 13 (18.3) |

COPD: Chronic obstructive pulmonary disease; Index date: day of the first procedure that led to the pathological diagnosis of NSCLC; NSCLC: Non-Small Cell Lung Cancer; NSAID: Non-Steroidal Anti-Inflammatory Drug.

^a^ More than one option could have been recorded per patient.

^b^ Comorbidities present in ≥4% of patients were listed.

^c^ Percentages were computed on patients with solid organ malignancy other than NSCLC (n = 19).
